# Examining the changing health care seeking behavior in the era of health sector reforms in India: evidences from the National Sample Surveys 2004 & 2014

**DOI:** 10.1186/s41256-017-0026-y

**Published:** 2017-03-06

**Authors:** Arnab Jana, Rounaq Basu

**Affiliations:** 1 Centre for Urban Science & Engineering, Indian Institute of Technology Bombay, Mumbai, Maharashtra 400076 India; 2 Intelligent Transportation Systems Lab, Department of Civil & Environmental Engineering, Massachusetts Institute of Technology, Cambridge, MA 02139 USA

**Keywords:** National health policies, Health care seeking behavior, Regional health status variation, NSS dataset, Health care in India

## Abstract

**Background:**

Health policy formulations in India have witnessed a shift from a reactive approach to a more proactive approach over the last decade. It is therefore important to understand the effectiveness of recent national health policies (such as the National Rural Health Mission and the National Urban Health Mission) in addressing the varied needs of the heterogeneous population of India.

**Methods:**

We use datasets from the National Sample Surveys carried out in 2004 and 2014 to understand the change in the health seeking behavior as a result of these policies. The choice of health care facilities and the associated expenditures are compared through descriptive analyses. A multinomial logistic regression is used to identify the significant parameters which contribute towards the share of health care providers in India. The health status of two economically disparate Indian states (Bihar and Kerala) are also compared through specific metrics of performance.

**Results:**

It is seen that due to increased availability of facilities in close proximity, both rural and urban residents prefer to avail of those facilities which will result in minimization of transportation cost. The effectiveness of national health policies is found to vary on a regional scale. Literacy and health status have a strong correlation, thereby reinforcing that Bihar still lags far behind Kerala in terms of access to equitable health care.

**Conclusion:**

Therefore, a hierarchical system, incorporating medical pluralism and tailor-made policies targeted at diverse health care demands, needs to be put in place to achieve Goal 3 of the Sustainable Development Goals as decreed by the United Nations, i.e., “health for all”.

## Background

Health policy formulations in India have witnessed a shift from a reactive to a more proactive approach over the last decade. The National Health Policy (NHP) 2002 focused on accessibility and availability issues in availing affordable health care [1]. It tried to address the polarization of health care infrastructure, medical personnel and other health resources in urban areas, which had led to augmented regional disparities in access to health care [2]. However, the widening economic, regional and gender disparities [3, 4] called for specific policies targeted at the urban and rural audiences separately. This led to the National Urban Health Mission (NUHM) and the National Rural Health Mission (NRHM) coming into being. These policies proposed implementation strategies to cater to the differential needs of the diverse socio-economic groups residing in the urban and rural areas of India respectively.

It is not surprising to find that individuals with greatest need for health care have the greatest difficulty in gaining access to health services and are least likely to have their health needs met [5]. India faces several obstacles in providing “health for all” (Goal 3 of the Sustainable Development Goals decreed by the United Nations), most notable of which are inadequate physical access to high-quality health services and dearth of qualified personnel at existing facilities [6]. These issues stem from underinvestment in the health sector. Only 4% of the GDP of India was invested in total health expenditure for the period 2011–2015, out of which only 1.3% was for the public health sector [7]. These figures are significantly lower when compared to the investment made by developed countries like USA and Japan. Patil AV, Somasundaram KV and Goyal RC [8] makes the following argument about this issue.

“*The lack of commitment to provide health care for its citizens is reflected in the inadequacy of the health infrastructure and low levels of financing, and also in declining support for the various healthcare demands of the people; especially since the 1980s, when the process of liberalization and opening up of the Indian economy to the world markets began.*”

With a change in objectives of policy-making and implementation of new health care policies in India over the last decade, we expect the health seeking behavior of Indian residents to have changed significantly. This research aims to examine and quantify these changes in terms of health care provider choice, associated expenditures and factors contributing to the choice. The analysis carried out as a part of this study will aid in understanding the effectiveness of recent health policies such as the NUHM and NRHM in addressing the varied needs of the heterogeneous population of India. Since there are cases of economic disparity among different states of India [9], we expect that their health statuses might be correlated with their economic statuses. Therefore, we compare the health statuses of two economically disparate states – Kerala (a high-income state) and Bihar (a low-income state). Such an examination is expected to provide insights regarding whether the effectiveness of national health policies is constant on a regional scale. The outcomes from this study might lead to formulation of strategies for implementation of NHP 2015 aiming towards all-dimensional shaping of health systems through encouragement of medical pluralism [10].

### Review of health policies in the post-2000 era

The NHP 1983 was aimed towards developing “*universal, comprehensive primary health care services which are relevant to the actual needs and priorities of the community at a cost which people can afford*”. Although the key objectives were de-centralization and de-professionalization of health care services, community participation did not work out as expected. Duggal R [11] and Nundy M [12] have outlined the following observations in order to highlight the gap in fulfilment of people’s needs: (i) Setting up of rural health care facilities to facilitate curative care was slow, thereby leading to overcrowding at urban facilities. (ii) There were severe issues with respect to quality and breadth of services available at both outpatient and inpatient facilities in public health care centers, which compelled patients to seek private facilities. (iii) A lack of proper medical training among health care providers was reported.

Eventually, the Ministry of Health and Family Welfare (MoHFW), Government of India drafted the NHP 2002 in order to address the gaps and fallouts of the NHP 1983. This policy emphasized the need to increase the overall utilization of public health care facilities. To trigger increase in the service delivery outlet, the government also encouraged private investment in health care. However, there were undesirable consequences of rising costs, increasing inequity and consumer exploitation associated with the private system of health care delivery [13]. In order to uplift the health care sector along with the country’s economic and social development, the Government of India launched the NRHM [14] and the NUHM [15].

Several management issues such as high absenteeism among staff at primary health centers (PHCs) and lack of training for effective service delivery in rural areas have been pointed out by Rao KS [16]. In order to reduce the growing disparity between urban and rural populations, NRHM was devised to improve the health service delivery in rural areas. The main aim of NRHM was “*to provide accessible, affordable, accountable, effective and reliable primary health care*” and to bridge the gap in rural health care through creation of Accredited Social Health Activists (ASHA). The mission entailed providing united funds to enable local planning and strengthening existing PHCs and community health centers (CHCs).

Researchers have also pointed out the decline in outpatient ratio at public health care centers over the past few decades, and further criticized that limited resources at these facilities are compelling patients to look for alternatives mostly in the form of private outlets and thereby incur catastrophic out-of-pocket (OOP) expenditures [17]. Such excessive OOP expenditures often lead to economic hardship and it has been found that the poor are affected the most in this regard [18]. Berman P, Ahuja R and Bhandari L [19] reported that the effect of OOP expenditure has significantly more impoverishing effect due to higher outpatient care needs in both urban and rural settings. In this context, it is also necessary to mention that price of the health care service, income of health care seekers and distance to the health care facility have been reported to be significant parameters in determining the choice of facility; however, the demand was reported to be price and income inelastic in rural India [20].

Balarajan Y, Selvaraj S and Subramanian SV [6] reported OOP to be one of the major causes of India’s population slipping below the poverty line. To diminish this severe burden on the meagre economic resources of economically weaker sections, ‘Rashtriya Swasthya Bima Yojana’ (RSBY) or ‘National Health Insurance Scheme’ was launched by the Ministry of Labor and Employment, Government of India. The scheme aimed to provide medical insurance to the marginalized classes and offered cashless hospitalization for up to INR 30,000. However, the inability of RSBY to cover outpatient care, which is the main contributor to OOP expenses, coupled with an outdated definition of ‘below the poverty line’ led to a fall in the effective outreach of the program as per its intended objectives [21]. It was concretely concluded by Narayana D [22] that the RSBY is far from achieving full penetration on a national scale, thereby making it essential for future policies and interventions to target the economically weaker sections.

NUHM was launched in 2012 in order to uplift the urban poor by providing them access to basic health care facilities. NUHM is set to cover India’s 7 big metropolitan areas and 772 cities with a population of more than 50,000 people each. The investment plan entails allocation of more than INR 225 billion to the health care sector reforms [23]. The ‘Urban Healthcare Delivery Model’ was devised in order to match the designed health service to the target segment. The service delivery model is a resource allocation model based on the target group of population to be served, specifying the human resources and level of service to be linked hierarchically. Since this is a relatively new initiative, the impact and effectiveness of this policy has yet to be examined.

## Data and Methods

The National Sample Surveys (NSS) are conducted through household interviews, using a random sample of households covering practically the entire geographical area of the country. Several studies have used NSS data to carry out varied analyses pertaining to the health care sector over the last decade [4, 6, 17, 19, 24–31]. This research makes use of the following two datasets for analysis: (1) Social Consumption: Health, NSS, 71st Round (January - June 2014), and (2) Morbidity and Health Care: NSS, 60th Round (January - June 2004). The 2004 survey focused on covering three main aspects: (i) Morbidity and utilization of health care services including immunization and maternal care, (ii) Problems of aged persons, and (iii) Expenditure of the households for availing the health care services. The 2014 survey included an additional focus on alternative schools of medicine with respect to prevalence of use, cost of treatment and type of ailments covered. It also concentrated on aspects of the condition of the elderly (60+) population which affected their state of health, economic independence and degree of isolation. A stratified multi-stage sample design was adopted along with referrals from the 2011 Census. The reader is requested to refer to Appendix C of the report ‘Key Indicators of Social Consumption in India: Health’ for further details on the sample design and estimation procedure adopted for this survey.

We explore the above-mentioned datasets on a national level so as to draw out major differences through our analyses. Since India has been host to traditional methods of medical treatment in the past, the differences in the choice of medical system are explored first on a sectoral basis (urban and rural). The choice of outpatient health care facility and the associated expenditures are also compared through descriptive analyses. This is followed by a discrete choice model to identify the significant parameters which contribute towards the share of outpatient health care providers in India. The state-wise comparison is carried out by isolating the data for the two states (Kerala and Bihar) and estimating the performance metrics for pairwise comparison.

## Results

### Change in health seeking behavior: 2004 to 2014

#### Choice of medical system in urban and rural India

The NSS 2004 survey did not contain questions pertinent to identifying the medical systems preferred by people. This was rectified by the NSS 2014 survey, analysis of which revealed around 90% of both urban and rural residents displaying a higher inclination towards allopathy treatment. The use of ‘*Other*’ systems such as AYUSH (Ayurveda, Yoga or Naturopathy Unani, Siddha and Homoeopathy) was reported to be around five to seven percent in both urban and rural areas. The competence and medical training of AYUSH medical officers has been found to be inadequate by Rao KD, Sundararaman T, Bhatnagar A, Gupta G, Kokho P and Jain K [32]. Therefore, it is interesting to note higher usage (difference of 1.5%) of such ‘*Other*’ treatment by urban males than their rural counterparts while less usage of the same (difference of 0.8%) was observed for urban females as compared to rural females. Moreover, the proportion of un-treated ailments was higher in rural areas (4%) as compared to urban areas (around 2.5%), irrespective of gender.

#### Choice of health care providers

The NSS 2014 survey involved a detailed questionnaire with respect to the divisions in the choice of facility unlike the 2004 survey, and therefore comparisons can only be made at an aggregate level. Table 1 highlights the change in choice of different health facilities for outpatient cases. Urban areas have witnessed a polarization of public health services over the last decade, which has subsequently led to a rise in the choice of public facilities by urban residents. New health facilities at the grassroots level have come up as part of NUHM and NRHM, leading to greater awareness of health care problems and the desire to avail of health care services. Therefore, it is also noteworthy that there has been an increase in the share of rural residents availing public health care. Due to increased participation of the private sector in health care, it is not surprising to see that the medical expenditure for non-hospitalized cases, i.e., outpatient cases, has increased by 104% over the past 10 years. Rates of hospitalization have also gone up across all age groups in both rural and urban sectors for both genders.

**Table 1:** Choice of different facilities for outpatient health care in 2014 (and 2004)^a^

Facility Type	Description	Urban		Rural	
		Male	Female	Male	Female
Public	Primary health care^b^	3.5	4.2	10.6	12.3
	Public Hospital	17.4	17.3	15.9	17.5
	Total Share	20.9 (19.2)	21.5 (19.2)	26.5 (21.7)	29.8 (22.9)
Private	Private Doctor/Clinic	48.9	50.8	52.7	48.9
	Private Hospital	30.2	27.7	20.8	21.3
	Total Share	79.1 (80.8)	78.5 (80.8)	73.5 (78.3)	70.2 (77.1)

^a^The values in parentheses are for 2004 while all other values are for 2014

^b^This segment includes all primary health care options: *HSC* Health Sub Center, *PHC* Primary Health Center, *ANM* Auxiliary Nurse Midwife, *ASHA* Accredited Social Health Activist, *AWW*, Anganwadi Worker dispensary, *CHC* Community Health Center, and *MMU* Mobile Medical Unit . All of these are part of the government-funded public health system in India. For further details, the reader is requested to refer to Appendix B of the report ‘Key Indicators of Social Consumption in India: Health’

An increase of 23% was observed for rural females reporting ailments during the last 15 days from the date of the survey. Moreover, they experienced an increase of 55% in the hospitalization rates, which is the highest among other gender-sector categories. Along with mobility constraints faced by Indian women in accessing health services, it has also been observed that Indian women frequently underreport illnesses [33]. Moreover, gender inequality in the health care system in India (only 6% female doctors in rural areas) has negatively affected women wishing to avail health care [34]. Therefore, this rise in hospitalization may be attributed to a rise in literacy levels and numerous attempts of the Government in the last decade to promote well-being of females, especially in rural areas (such as NRHM). However, one must note that the proportion of female patients availing private facilities is lower than their male counterparts for both urban and rural regions, which highlights the gender inequality in the Indian social construct.

#### Factors affecting share of health care providers in 2014

Since the 2014 dataset offers us several alternatives in terms of outpatient health care providers, we utilize the heterogeneity in examining the factors which drive the share of these facilities among the Indian populace. ‘*HSC/ANM/ASHA/AWW*’ (represented as *PUB01*) accounted for only 1 and 2% of the urban and rural samples respectively, and was therefore discarded for further analysis. The other alternatives as given in the NSS 2014 questionnaire are ‘*PHC/Dispensary/CHC/MMU*’ (represented as *PUB02*), ‘*Private Doctor/Clinic*’ (represented as *PVT01*), ‘*Private Hospital*’ (represented as *PVT02*), ‘*Self/Household member/Friend*’ (represented as *OTH01*) and ‘*Medicine shop and others*’ (represented as *OTH02*). ‘*Public Hospital*’ (*PUB03* – 18% share) has been taken as the reference alternative.

Descriptive statistics (mean, and standard deviation within parentheses) for the independent variables considered for the choice model in this paper have been presented in Table 2. It is noteworthy that rural residents bore lesser medical expenses than their urban counterparts, whereas the opposite trend was observed for transport expenses. Such statistics reveal the gap in spatial accessibility to health care facilities faced by the rural population. Higher mean household size and lower mean monthly household expenditure are also observed for rural residents, as expected. Another interesting observation is that the mean literacy rate is higher in rural areas as compared to urban areas, highlighting the migratory tendencies of the labor class.

**Table 2:** Descriptive statistics for independent variables^a^

Variable	Description	Urban		Rural	
		Mean	Std. Dev.	Mean	Std. Dev.
MEDEXP	Medical expenditure (INR)	758	2,381	609	1,659
TRNEXP	Transport expenditure (INR)	55	325	65	243
AGE	Age (years)	41	24	38	24
HHSZ	Household size	5	3	6	3
HHEXP	Monthly household expenditure (INR)	12,221	8,948	8,244	5,251
BM1501^b^	1 if ‘Ailment started more than 15 days ago and is continuing’; otherwise 2	1.35	-	1.44	-
BM1502^b^	1 if ‘Ailment started more than 15 days ago and has ended’; otherwise 2	1.96	-	1.94	-
BL1501^b^	1 if ‘Ailment started less than 15 days ago and is continuing’; otherwise 2	1.89	-	1.88	-
MALE^b^	1 if ‘Male’; otherwise 2	1.54	-	1.53	-
BINMRD^b^	1 if ‘Married’; otherwise 2	1.41	-	1.43	-
LITERT^b^	1 if ‘Literate’; otherwise 2	1.30	-	1.47	-
BINSCH^b^	1 if ‘Covered by any scheme for health expenditure support’; otherwise 2	1.76	-	1.80	-
BINCHR^b^	1 if ‘Chronic ailment’; otherwise 2	1.42	-	1.51	-

^a^It should be noted that standard deviation is not reported for binary variables

^b^Binary variables

Due to socio-economic and geographical disparities between urban and rural dwellers, we thought it pertinent to model their health care seeking behavior differently and unsurprisingly, there were considerable differences. Discrete choice models are used to determine the choices made by decision-making units (such as individuals or organizations) among a finite set of alternatives [35]. The multinomial logistic regression model (referred to as MNL) is the most widely applied one for scenarios with more than two possible discrete outcomes [36]. A short description of the multinomial logistic regression model is provided as follows.

The MNL model is based on the theory of maximizing utility, where $\mathrm{Utility}\;\left(\mathrm{U}\right)= Systematic\; component\;(V)+ Random\; component\;\left(\varepsilon \right)$. The probability of individual $n$ choosing alternative $i$ from the choice set ${C}_n$ is given by:

$$ P\left( i\Big|{C}_n\right)=\frac{e^{\mu {V}_{in}}}{{\displaystyle {\sum}_{j\in {C}_n}}{e}^{\mu {V}_{j n}}} $$

where μ is the scale parameter (usually considered as unity for ease of model estimation) and ${V}_{in}$ is the systematic component of the utility. It is assumed that the error terms (${\varepsilon}_{in}$) are independent and identically Gumbel-distributed (i.i.d.). This leads to the ‘independence from irrelevant alternatives’ (IIA) assumption, which implies that the probability of choosing one alternative over another does not depend on the presence or absence of other ‘irrelevant’ alternatives. The MNL models capturing the behavior of rural and urban residents are shown in Tables 3 and 4 respectively.

**Table 3:** MNL model for health care seeking behavior of rural dwellers in 2014^a^ ^b^

	PUB02	PVT01	PVT02	OTH01	OTH02
Intercept	0.376^***^ (3.163)	1.160^***^ (13.493)	−0.293^***^ (−2.920)	−0.555^***^ (−3.132)	0.891^***^ (7.404)
MEDEXP [Medical expenditure]	−0.001^***^ (−10.535)	3.34E-04^***^ (9.793)	4.08E-04^***^ (11.946)	−2.58E-04^**^ (−2.257)	−0.001^***^ (−9.565)
TRNEXP [Transport expenditure]	9.12E-05 (0.578)	−0.002^***^ (−11.559)	−0.001^***^ (−7.032)	−0.002^***^ (−3.245)	−0.011^***^ (−10.933)
AGE [Age]	4.65E-04 (0.267)	−0.007^***^ (−5.588)	−0.007^***^ (−4.774)	0.002 (0.858)	−0.002 (−0.885)
HHSZ [Household Size]	0.012 (0.771)	0.020^**^ (1.960)	−0.063^***^ (−5.593)	4.73E-04 (0.020)	0.050^***^ (3.297)
HHEXP [Household Expenditure]	−2.20E-05^***^ (−2.427)	2.68E-05^***^ (4.662)	7.58E-05^***^ (12.933)	−4.11E-05^***^ (−2.706)	−4.56E-05^***^ (−4.605)
BINCHR^c^ [Chronic ailment]	−0.167 (−1.213)	−0.174^**^ (−2.006)	0.185^*^ (1.939)	−1.164^***^ (−5.971)	−0.669^***^ (4.557)
BM1501^c^ [Ailment started more than 15 days ago and is continuing]	−0.895^***^ (−6.210)	−0.573^***^ (−5.960)	0.109 (0.986)	−0.601^***^ (−3.146)	−1.146^***^ (−7.764)
BM1502^c^ [Ailment started more than 15 days ago and has ended]	−0.053 (−0.393)	−0.390^***^ (−3.609)	−0.385^***^ (−2.760)	0.221 (1.287)	−0.418^***^ (−2.995)
BL1501^c^ [Ailment started less than 15 days ago and is continuing]	−0.276^**^ (−2.375)	−0.056 (−0.631)	0.075 (0.698)	−0.009 (−0.061)	−0.244^**^ (−2.236)
MALE^c^ [Gender is male]	−0.080 (−1.206)	−0.026 (−0.568)	0.015 (0.294)	−0.019 (−0.190)	−0.097 (−1.416)
BINMRD^c^ [Married]	−0.080 (−1.061)	0.062 (1.157)	0.201^***^ (3.421)	0.057 (0.474)	0.198^**^ (2.425)
LITERT^c^ [Literate]	0.173^**^ (2.541)	0.026 (0.531)	−0.028 (−0.531)	−0.051 (−0.497)	0.063 (0.894)
BINSCH^c^ [Covered by health care expenditure support scheme]	−0.038 (−0.468)	−0.259^***^ (−4.452)	0.229^***^ (3.804)	−0.129 (−0.981)	−0.059 (−0.695)

^*^significant at 90% confidence interval (*p* < 0.1); ^**^significant at 95% (*p* < 0.05); ^***^significant at 99% (*p* < 0.01)

^a^Numbers outside parentheses reflect parameter estimates, while numbers in parentheses reflect t-statistic values

^b^The reader is requested to refer to Table 2 for a more detailed description of variables

^c^Binary variables

**Table 4:** MNL model for health care seeking behavior of urban dwellers in 2014^a,^ ^b^

	PUB02	PVT01	PVT02	OTH01	OTH02
Intercept	−0.709^***^ (−3.790)	1.164^***^ (13.139)	0.229^**^ (2.318)	−1.421^***^ (−7.483)	0.343^**^ (2.287)
MEDEXP [Medical expenditure]	−0.001^***^ (−7.000)	2.08E-04^***^ (7.802)	2.38E-04^***^ (9.015)	−0.001^***^ (−4.523)	−0.001^***^ (−6.493)
TRNEXP [Transport expenditure]	−1.61E-06 (−0.005)	−0.001^***^ (−6.669)	−2.68E-04^***^ (−4.111)	−0.001^*^ (−1.673)	−0.019^***^ (−9.720)
AGE [Age]	0.007^***^ (2.772)	−0.004^***^ (−2.981)	−0.003^**^ (−2.525)	0.004 (1.241)	0.003 (1.293)
HHSZ [Household size]	0.014 (0.617)	−0.019^**^ (−2.051)	−0.075^***^ (−7.254)	0.022 (1.063)	−0.013 (−0.703)
HHEXP [Household expenditure]	−3.11E-05^***^ (−2.982)	4.80E-05^***^ (12.714)	5.25E-05^***^ (13.669)	3.64E-05^***^ (4.465)	−4.89E-06 (−0.629)
BINCHR^c^ [Chronic ailment]	−0.034 (−0.148)	−0.170^*^ (−1.933)	0.114 (1.222)	−1.165^***^ (−5.333)	−0.644^***^ (−3.371)
BM1501^c^ [Ailment started more than 15 days ago and is continuing]	−0.973^***^ (−4.049)	−0.668^***^ (−6.638)	−0.035 (−0.317)	−0.907^***^ (−4.175)	−1.472^***^ (−7.636)
BM1502^c^ [Ailment started more than 15 days ago and has ended]	−0.265 (−1.080)	−0.304^**^ (−2.427)	−0.319^**^ (−2.132)	−0.068 (−0.319)	−0.318^*^ (−1.799)
BL1501^c^ [Ailment started less than 15 days ago and is continuing]	−0.183 (−1.067)	−0.208^**^ (−2.271)	−0.260^**^ (−2.386)	−0.194 (−1.209)	−0.344^***^ (−2.713)
MALE^c^ [Gender is male]	−0.231^**^ (−2.255)	−0.173^***^ (−3.739)	−0.100^**^ (−2.030)	−0.051 (−0.470)	−0.131 (−1.592)
BINMRD^c^ [Married]	−0.329^***^ (−2.978)	0.006 (0.117)	0.111^**^ (2.003)	0.011 (0.088)	0.134 (1.393)
LITERT^c^ [Literate]	0.207^*^ (1.848)	0.053 (1.019)	−0.011 (−0.204)	0.271^**^ (2.166)	0.246^***^ (2.627)
BINSCH^c^ [Covered by health care expenditure support scheme]	0.093 (0.821)	−0.463^***^ (−8.655)	−0.159^***^ (−2.907)	−0.138 (−1.064)	−0.277^***^ (−2.761)

^*^significant at 90% confidence interval (*p* < 0.1);^**^significant at 95% (*p* < 0.05);^***^significant at 99% (*p* < 0.01)

^a^Numbers outside parentheses reflect parameter estimates, while numbers in parentheses reflect t-statistic values

^b^The reader is requested to refer to Table 2 for a more detailed description of variables

^c^Binary variables

An increase in medical expenditure marginally augments the probability of rural residents who choose private health care options, while diminishing the probabilities of other alternatives. Transport expenditure is seen to negatively affect non-public alternatives from Table 3. Younger rural dwellers are observed to prefer private facilities and medicine shops, while older residents prefer PHCs and self-treatment. Smaller household sizes seem to prefer private hospitals. As expected, private alternatives have households with higher monthly expenditures associated with them. Both people suffering from chronic ailments and people with ailments which were still continuing when the survey was taken were found to prefer private hospitals. The same trend was observed for rural males. In the case of *BM1502* ailments, the preference was for self-treatment or consulting a household member or friend. Married rural residents seemed to prefer PHCs the least, whereas the opposite behavior was observed for literate rural residents. Since private hospitals are associated with high medical expenditures, it was not surprising to see rural people covered by health expenditure support schemes having the maximum preference for such facilities.

From Table 4, medical expenditure is seen to affect the choice for different alternatives among urban residents in the same manner as their rural counterparts. However, due to increased availability of facilities, there is a negative effect of transport expenditure on the choice probabilities. Younger residents are seen to prefer private facilities more. Larger households are observed to prefer public PHCs and self-treatment. Although extremely marginal, it is interesting to note that households with large monthly expenditures preferred private facilities and self-treatment. Urban patients with chronic ailments exhibited the same behavior as their rural counterparts. Residents with all the three different types of ailments (*BM1501*, *BM1502* and *BL1501*) seem to prefer public hospital. The same behavior was observed for male residents. Married residents had the least preference for PHCs, which is consistent with the observation for rural dwellers. Surprisingly, literate urban dwellers displayed least preference for private hospitals. It is also noteworthy that people covered by health expenditure support schemes preferred public PHCs over all the other alternatives.

2014 witnessed a rise in reporting of illnesses and hospitalization rates, implying an improvement in accessibility of health care facilities. Although the proportion of population availing ‘do-nothing’ scenario has reduced, the mission of “health for all” is not successful until this proportion diminishes significantly.

### Inter-state variability of health care provisioning: Kerala vs Bihar

India’s socioeconomic diversity is exhibited through the sharp contrast between the states of Bihar and Kerala. While Kerala leads the literacy rate of the country (94%), Bihar has the lowest (61.8%). The same trend is exhibited for female literacy rates as well, according to the 2011 Census. Kerala, besides having the lowest mortality rate, is also far ahead of other states in the field of health care, as reflected by indicators like birth rate and life expectancy. Kerala, with an exceptionally advanced health care system in the country, had a hospital bed for every 325 persons (NSS 2004) and the results of the NSS 2014 survey indicate an exceptionally high proportion of persons receiving hospitalized treatment in the state. On the other hand, the poor performance of public hospitals in Bihar is revealed by the fact that use of public health care facilities for treatment of ailment was lowest in the rural areas of Bihar (5%) compared to other states. Although medical expenditure for treated ailments was lowest in Kerala for both the rural and urban sector, the highest expenditure for public sector hospitals was reported from urban Bihar. Such contrasting statistics form the basis of a separate comparative examination of the health seeking behavior in Kerala and Bihar.

48.8% residents of Bihar who felt that required specific services were not available at government facilities and thereby availed of a private doctor/clinic were from the urban sector, as compared to 39.8% for Kerala. A difference of 3% in favor of urban Kerala was seen again for those who availed of private hospitals for the same reason. 68.6% of Bihar residents vis-à-vis just 51.3% (Pearson Chi-Square and Likelihood Ratio significant at 95%) of Keralites who found the quality of health care at government facilities to be unsatisfactory and visited a private doctor/clinic were from the rural sector. Considering the same reason, the proportions visiting private hospital change to 77.6% for rural Kerala and 49.2% for rural Bihar (Pearson Chi-Square and Likelihood Ratio significant at 95%). A difference of 55% (Pearson Chi-Square and Likelihood Ratio significant at 95%) was seen in favor of rural Kerala for patients visiting private doctor/clinic because the government facilities, although having satisfactory quality, were too far away. This increases to 60.3% when the same reason is considered for visiting private hospitals from the rural sector. 63.6% of Bihar residents who visited private hospitals because the government facilities, although having satisfactory quality, involved long waiting times were from the rural sector, as compared to 45.3% for Kerala.

This analysis shows that both the rural and urban sectors of Bihar lag far behind Kerala in terms of public health care delivery. It can be clearly seen that rural Bihar needs a major upheaval in the health care sector in terms of provisioning, medical personnel and policy interventions ensuring quality and quantity. The lack of proper provisioning of facilities and inadequacy in providing quality services at an affordable rate has led to such a gulf between Kerala and Bihar.

## Discussion & policy implications

The results show that there has been considerable improvement in terms of accessibility and availability of health care services over the last decade. Although improved quality of services at public facilities induced a shift in favor of them, private facilities continue to dominate the health care service provider market in India. It is observed that younger population majorly opted for private facilities in the event of illness. It has been pointed out by Ergler CR, Sakdapolrak P, Bohle H-G and Kearns RA [37] that affordability and physical access to health care in less developed countries should also include a focus on emotional dimensions of utilization. In general, private facilities offer lesser waiting times and speedier service delivery which might have led to their popularity. Impedances to avail health care often lead to dependency. Altruistic behavior, although observed in India, often has considerable delays associated with it. Availability of companions often govern the choice, especially to counter impedances such as delay, restricted mobility, travel experience and loneliness. In case of smaller households in rural areas, especially for couples, these impedances are often alleviated by visiting private hospitals.

Despite the increased presence of public facilities in rural areas over the last decade, there is a dearth in the variety and quality of services offered by them. Chronic or long-term ailments require specialized care and trained medical personnel, which are often lacking at such facilities leaving these patients no other option but to avail private hospitals. The case improves for public hospitals in urban areas, with an upgrade in the range and quality of available health care services. However, the state of health in India has a high variability among states.

From the state-level analysis of Bihar and Kerala, literacy and health status were found to have a strong correlation. Increased perception and awareness about different illnesses is associated with rise in literacy, along with augmented desire to utilize the available health care options. Moreover, cognizance about one’s health status is also directly proportional to literacy level. Therefore, a larger sample was obtained from Kerala who could report about their health care needs and behavior. Kerala, having the highest literacy rate in India, also has the strongest health care system in the country. Despite showing improvement in the health care service delivery sector from 2004, Bihar still lags far behind Kerala. This gulf needs to be minimized through strong public-private partnerships to ensure equitable access. Moreover, encouraging private investment might result in successful implementation of public programs.

Existing health policies have not yet incorporated the widely seen phenomenon of medical pluralism, which is why the National Health Policy 2015 has listed that as one of its critical areas of focus. Therefore, a hierarchical system incorporating medical pluralism needs to be put in place so that the heterogeneous health needs of the population are met through facilities offering a diverse range of services. For successful sustainability of this system, quality monitoring mechanisms must be put in place. Such mechanisms are important because of the presence of informal health markets in marginalized regions. George A and Iyer A [38] have highlighted the dependency of such providers on the government and private sector for training and referral networks. Therefore, future health policies should try to ensure that all facilities offer acceptable services that meet a certain critical quality benchmark, so that medical expenditure becomes the major differentiating factor between such facilities.

## Limitations

Since this research considers cross-sectional surveys as datasets for analysis, there are a few concerns that need to be kept in mind while interpreting the results. Such surveys usually need to be mindful of the following issues: (i) choosing a representative sample, (ii) sample size, (iii) specific inclusion and exclusion criteria at the design stage, (iv) potential bias due to non-response, and (v) potential misclassification due to recall bias. While the authors acknowledge all of the aforementioned issues, there is no other national survey in India that provides such a large dataset on these topics. Moreover, the survey is designed and conducted by the government, and claims to consider a representative sample. An additional limitation of this research is that the MNL model only identifies the statistically significant variables influencing the choice of health care service provider, but cannot establish a causal relationship. Future research efforts may be directed towards developing structural equation models that are able to establish causality.

## Conclusion

In the last decade, two major policy interventions have been formulated and implemented in India under NHP 2002, namely NUHM and NRHM. These policies were backed up by RSBY, enabling disadvantaged groups to access quality health care as per their needs. In the current context, NHP 2015 is being drafted to bring in a holistic yet deterministic improvement of health status of the people in general. Both NUHM and NRHM exhibited a systems approach towards improvement of health care availability and accessibility. Effects of such policies clearly show positive outcomes such as increase in reported illnesses and seeking of treatment based on medical advice. However, their effectiveness is found to vary on a regional basis depending on the economic and literacy status of the state. This research clearly outlines the need for public-private partnership projects in the health care sector. These ventures should be able to overcome the dual obstacles of service availability (as compared to public projects) and affordability (as compared to private projects). In the near future, integration of providers in terms of referral system sensitive to the socio-economic heterogeneity of the nation will be of paramount importance to ensure acceptance of the facilities, considering the pluralities of the health care system practiced in India.

## Abbreviations

AYUSH: Ayurveda, Yoga or Naturopathy Unani, Siddha and Homoeopathy
CHC: Community health centre
MNL: Multinomial logistic regression
MoHFW: Ministry of health & family welfare
NHP: National health policy
NRHM: National rural health mission
NSS: National sample surveys
NUHM: National urban health mission
OOP: Out-of-pocket
PHC: Primary health centre
RSBY: Rashtriya Swasthya Bima Yojana
